# An EEG dataset to study neural correlates of audiovisual long-term memory retrieval

**DOI:** 10.1038/s41597-025-06203-1

**Published:** 2025-12-08

**Authors:** Ana Matran-Fernandez, Sebastian Halder

**Affiliations:** Brain-Computer Interfaces and Neural Engineering Lab, School of Computer Science and Electronic Engineering, University of Essex, Colchester, UK

**Keywords:** Cognitive neuroscience, Biomedical engineering, Human behaviour

## Abstract

Memory retrieval is a fundamental cognitive process that plays a critical role in our lives. Studying the neural correlates of this process has significant implications for numerous fields, such as education and health care. Advances in neuroimaging technologies have facilitated the use of neural data, such as electroencephalography (EEG), to decode cognitive states associated with memory tasks. However, most memory research is still conducted using simple stimuli, such as lists of words, and it is unclear how much the discoveries made with such stimuli generalise to more naturalistic scenarios. We introduce a dataset of EEG signals from 27 participants acquired while they watched 10-second long clips of movies (some of which they had previously seen), together with annotations that reflect whether they recognised or remembered the scenes and the time points of recognition. This dataset allows the study of neural correlates of long-term memory recall in a naturalistic task.

## Background & Summary

Memory retrieval is a cognitive process that plays a critical role in human experience, influencing learning, decision-making, and emotional regulation^1^. Understanding the neural mechanisms underlying memory recall is a critical area of cognitive neuroscience, with implications for many domains, including education and healthcare^2^. Memory recall processes involve complex interactions between brain regions, including the hippocampus, prefrontal cortex, and sensory cortices, which are influenced by emotional and contextual factors^3^. Several neuroimaging modalities and techniques can be used to study these processes^4^. For example, functional magnetic resonance imaging (fMRI) has been widely used to investigate memory, offering high spatial resolution that allows for the identification of specific brain regions involved in memory processes^4–6^ and even showing shared neural activity patterns across individuals when recalling a common event^7^. Electroencephalography (EEG), on the other hand, has the advantage of having a high temporal resolution, so that it can capture dynamic brain activity during memory tasks^8–10^. Research using EEG has revealed specific spectral patterns associated with memory retrieval, such as increased theta power and decreased alpha power during episodic recall^9,11–15^. Event-related potentials have also been linked to successful memory encoding and recall^16,17^.

Despite these insights, there is limited research on how EEG signals reflect the neural dynamics of recalling audiovisual stimuli, which tend to elicit more dynamic brain responses than the type of stimuli typically used in memory research studies^18,19^. This is important because activation of memory subcomponents reflects the brain regions that were engaged during encoding^7,20,21^, which in turn depend on the modality of the stimulus used^22,23^. Since most memories are complex and multimodal, it remains to be seen whether the knowledge acquired with simple stimuli, such as lists of words or images^23–27^, generalises to more naturalistic scenarios. Audiovisual stimuli offer a unique advantage in memory research by simulating real-world experiences through multimodal integration. Previous studies have shown that audiovisual cues enhance memory encoding and recall compared to unimodal stimuli, as they engage multiple sensory pathways and emotional processing centres in the brain^7,20,21^. Additionally, movies provide a controlled yet ecologically valid medium for studying memory recall, enabling researchers to investigate how continuous dynamic stimuli are segmented into discrete episodic events^28–30^. However, the neural correlates of memory recall for familiar audiovisual stimuli remain under-explored.

In this paper, we describe a dataset of EEG signals collected to investigate the neural correlates of memory recall using familiar audiovisual stimuli. Participants were presented 10-second fragments of movies they had previously seen (and some from movies they had not), ensuring familiarity with the content and organically offering a way to study long-term memory without controlling the encoding phase. EEG data were recorded while participants recalled these movie fragments, providing a rich dataset for analysing brain activity during episodic memory retrieval.

This dataset has significant potential for advancing research on memory recall mechanisms. It can be used to explore the role of different brain regions in processing familiar audiovisual stimuli and to develop computational models for detecting successful memory retrieval. Additionally, it may contribute to applications in neurorehabilitation and brain-computer interfaces aimed at evaluating and enhancing memory function in clinical populations.

## Methods

Data acquisition took place at two different time points, in Summer 2019 and Spring 2024. The time lapse between the two cohorts of participants was due to COVID and lack of funding. Participants were recruited through word of mouth and a university-wide email list. Everyone who expressed their interest in the experiment was sent an information sheet describing the protocol. Ethical approval was obtained from the University of Essex’s ethics committee for each of the cohorts (ETH1819-0250 for 2019; ETH2324-0873 for 2024). All participants read and signed an informed consent form (in which they agreed to both take part in the experiment and have their data shared after anonymisation) before data collection started.

The experiment was conducted in two phases for each participant. First, prior to being invited to EEG data acquisition, participants were sent a link to an online survey hosted in *Qualtrics*. The survey consisted of four parts (see Survey Section) and was designed to identify which movies from our set had been watched previously by the participants. This allowed us to construct a subject-specific set of stimuli that balanced the number of clips that the participant was expected to remember vs. the ones they were not. All participants from the 2019 cohort that filled the survey were invited to the EEG data acquisition phase of the experiment. However, after early analyses of the behavioural data from this cohort, for the 2024 data acquisition we excluded those volunteers who had watched less than 30 out of our set of 93 movies according to their answers to the survey. This was done in an attempt to create a more balanced dataset of EEG signals for clips remembered and not remembered (more on this below).

The second phase was EEG data acquisition. Participants were seated comfortably in a room free from noise and distractions in front of the computer displaying the stimuli. After placing the EEG cap and sensors, they read the experiment instructions from the screen in front of them. Participants were given two guided trials as practice to become familiar with the mechanics of the experiment. In these, the experimenter asked them to select specific options so that they would experience the sequence of questions and actions for each clip. We provide full details for this phase in the EEG Experimental Protocol Section.

### Survey

The survey administered to participants prior to EEG data collection had four different parts, which we detail here.

#### Participant demographics

Participants were first asked about their age, gender (male/female/non-binary), and preferred hand (left/right). The survey then moved on to the second part.

#### Movie annotations

**Fig. 1:**
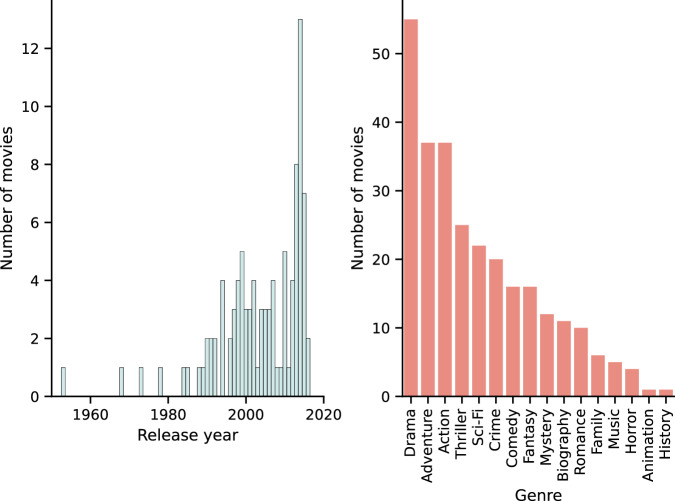
Distributions of (left) release years and (right) genres for the movies in the Cohendet movie dataset. The genres were extracted from IMDb; note that each movie can belong to more than one genre.

The second section of the survey followed the design by Cohendet *et al*. to create the memorability annotations for the clips for each participant^31^. Figure 1 shows the distributions of release years and genres that are included in Cohendet’s movie dataset.

Briefly, participants were sequentially shown the title and movie poster of each of the movies from which the clips had been extracted. They were first asked whether they had ever watched the movie (possible answers: “Yes”, “No”). If they answered positively, they were also asked when was the last time they had watched it (possible answers: “less than a month ago”, “1 month to 1 year ago”, “1–5 years ago”, “5–10 years ago”, or “more than 10 years ago”) and how many times (possible answers: “once”, “2–4”, “5–9”, or “more than 10”). For the movies participants had not watched, they were only asked how confident they were that they had not watched them using a slider (0 = not confident; 100 = very confident).

#### Movie-viewing behaviours

The third part of the survey asked participants about their movie-viewing behaviours and preferences.

Participants were asked how often they watch films on different media (e.g., TV, streaming, cinema...) and their favourite film genres (selected from a list of options). Additionally, given different forms of art and entertainment (e.g., film, TV, literature), we asked participants to choose the word/s that best described their experience of each. For example, they might find music “relaxing” and “emotional”, or literature a form of “escapism”. The full list of questions, together with the set of possible answers for each is available with the dataset^32^ (see Data Records Section).

This survey question is asking you to think about different forms of art and entertainment—such as film, TV, theatre/dance, literature, video games, music, and sports—and choose the words that best describe your experience of each. For example, you might find music “relaxing” and “emotional,” video games “exciting” and “sociable,” or literature “rewarding” and “escapism.” You can select multiple words for each category.

#### Prospective and Retrospective Memory Questionnaire

The final part of the survey was used to measure general memory from our participants. We used a subset of 10 items (items 1–4, 6, 8–10, 15 and 16) from the Prospective and Retrospective Memory Questionnaire (PRMQ)^33^. We excluded items from the “prospective long-term” subscales (items 5, 7, 12, and 14), as well as item 12 as it was not relevant to our experiment. Item 13 was accidentally left out.

According to the original PRQM design, participants reported how often they made different types of memory errors using a 5-point scale (1 = never; 5 = very often).

### Movie Stimuli

The pool of stimuli used in the EEG acquisition phase of the experiment consisted of 660 10-second-long clips from 93 feature films, which were annotated by Cohendet *et al*. in terms of their likelihood to be remembered^31^.

Clips were originally categorised as either “neutral” or “typical”. “Neutral” clips contained scenes that are not easily memorable for a given movie (e.g., a panoramic view of a forest). Instead, “typical” clips were chosen to contain parts of the movie that are likely to be remembered by someone who has watched it (e.g., a fight between the protagonist and the antagonist).

The original memorability annotations indicated the probability that someone who has watched the movie would remember the specific clip (range = 0–1).

Since our study relied on the memorability annotations from the clips, the construction of the participant-specific stimuli for our experiments was based on an algorithm that follows a similar paradigm to^31^, but which differed between the 2019 and 2024 cohorts, as we explain below.

#### Stimuli generation for the 2019 cohort

Participants from the 2019 cohort had watched, on average = 41.6 ± 16.9 (range = 15–70) movies from the dataset.

Each participant was shown two clips from each of the movies they had seen (one from the “typical” and one from the “neutral” categories), plus a random selection of clips with low and high memorability scores from the movies they had not watched.

This method for clip selection resulted in a relatively imbalanced dataset for some participants who had not watched sufficient movies, with higher number of EEG epochs being “not remembered” than “remembered”. This makes it difficult to explore the neural correlates of memory recall as intended. For this reason, we added an exclusion criteria prior to restarting data collection in 2024.

#### Stimuli generation for the 2024 cohort

For the 2024 cohort we excluded participants who reported having watched less than 30 movies from our list when filling out the online survey. Four participants were excluded for this reason and did not proceed to the EEG experiment; the average number of movies watched by those who attended the EEG experiment was 51 ± 13.6 (range = 34–73 movies).

For the 2024 cohort the list of stimuli was constructed using a linear regressor. The model was trained using the behavioural data from the 2019 cohort and regularly updated as more data became available. The regressor predicted the probability that a specific participant would remember each clip of the database given as inputs the participant’s answers to the survey (if they had watched the movie, how many times, and when last) and the clip’s memorability score.

Although the study was conducted in two phases (2019 and 2024), the experimental setup, task instructions, and participant experience were identical across both cohorts. The only difference was this minor refinement to the stimulus selection procedure in 2024. Crucially, this change did not alter the nature of the task from the participant’s perspective, and behavioural validation analyses (see Technical Validation Section) show comparable distributions of recognized vs. unrecognized trials across the two cohorts. Accordingly, the 2019 and 2024 data should be considered as part of the same experiment rather than as separate pilot and main versions.

### EEG Experimental Protocol

The experimental protocol for EEG data collection was explained to participants before data collection began through written instructions shown on the screen at the beginning of the experiment, including 2 guided practice trials to ensure they understood how to respond.

The initial written instructions aimed to provide clarity to participants regarding the difference between “recognising” and “remembering” a clip, as follows:

*After each movie clip, we will first ask you whether you recognise the movie. This means whether you know what movie it is from, regardless of whether you have watched that movie*.

*Then we will ask you if you remember that specific scene. That means whether you remember specifically when in the plot of the movie the clip took place*.

*You are NOT asked to guess whether a video comes from a movie whose title you saw in the questionnaire, but whether you remember having watched that specific clip*.

**Fig. 2:**
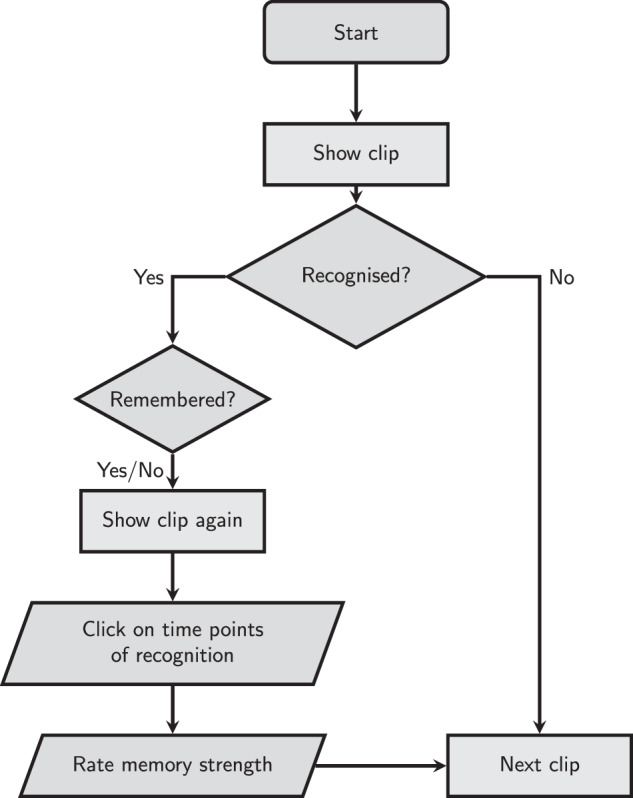
Flowchart of the recognition and memory task sequence for one clip. Participants begin the task by viewing a video clip. They are then asked whether they recognised the content. If not, the experiment proceeds to the next clip. If they do recognise it, they are further asked whether they remember it. Regardless of the answer, the clip is shown again, and participants are prompted to mark the time points at which recognition occurred. Finally, they rate the strength of their memory before proceeding to the next clip. All clips last 10 seconds. The experiment does not advance from one decision point to the next until the participant provides an answer (there are no time limits).

The clips selected for a participant were shown in randomised order. The sequence for each clip is illustrated in Fig. 2. After a new clip was shown, they were asked whether they recognised the movie it belonged to (regardless of whether they had watched it; “Do you know this movie?”). If they did not (right mouse click), the clip was labelled as “not recognised” and the next clip was shown. If they answered yes (left mouse click), they were asked whether they remembered watching it previously (i.e., whether they remember specifically watching the scene and when in the plot of the movie it took place; “Do you remember watching this clip previously?”). If a participant answered positively, the clip was labelled as “remembered”. Clips which were recognised by participants but not remembered were labelled as “recognised”.

When a clip was either “recognised” or “remembered”, participants watched it a second time. They were asked to click the left mouse button to mark the time points (as many times as they wanted) that caused them to recognise the movie (the instruction on the screen was “Click the mouse when you recognise something”). For these clips, we also asked participants to use the mouse’s scroll wheel to report how strong their memory of the clip was (on a scale of 1–5; “How intense was your memory of this clip?”) after the second watch. In the instructions, the experimenters specified that this question referred not only to how vividly they remembered the clip, but could also include aspects such as how well they remembered the experience of watching the clip. For example, whether they remembered who they watched it with, where they were, and how they felt.

After a brief pause (1 second long), the next clip started playing. Every ten clips, participants were offered a break (the instructions on the screen were “BREAK -- Left click when you're ready to continue”), and they could choose when to continue by clicking on the left mouse button to continue the experiment.

### EEG Data Collection

The protocol and equipment used for data collection did not change between the 2019 and 2024 cohorts (other than the changes already mentioned to select the participant-specific clips). The first cohort contains data from 12 participants (6 females, 6 males; one left-handed; age range = 21–47, mean age  = 29.25 ± 6.49 years old) and the second 15 participants (2 females, 13 males; one left-handed; age range = 22–34, mean age  = 26.8 ± 3.76 years old). Demographic information from all participants is given in Table 1. All participants had normal or corrected-to-normal vision. Participants were paid for their time.

**Table 1:** Participant information for our dataset.

Participant	Cohort	Age	Sex	Handedness	Number of movies watched
1	2019	25	male	right	28
2	2019	24	female	right	15
3	2019	47	female	right	33
4	2019	26	female	right	35
5	2019	29	male	right	33
6	2019	21	male	right	32
7	2019	29	female	right	61
8	2019	31	female	right	59
9	2019	31	male	right	54
10	2019	30	male	right	65
11	2019	26	female	right	32
12	2019	32	male	left	70
13	2024	28	male	right	51
14	2024	29	male	right	38
15	2024	23	female	right	55
16	2024	29	female	right	37
17	2024	33	male	right	47
18	2024	25	male	left	54
19	2024	29	male	right	34
20	2024	30	male	right	66
21	2024	23	male	right	34
22	2024	27	male	right	68
23	2024	23	male	right	73
24	2024	25	male	right	45
25	2024	34	male	right	72
26	2024	22	male	right	40
27	2024	22	male	right	55

For each participant, we report when the data were acquired, the age, self-reported sex, and handedness of the participant, and the number of movies from the annotated dataset they reported watching prior to EEG data acquisition.

Electroencephalography data were collected using a 64-channel BioSemi ActiveTwo recording system at a sampling rate of 2048 Hz. Electrodes were placed according to the international 10–20 system.

Synchronisation between EEG and computer stimuli was achieved by sending triggers from the experimental computer at the onset of each event.

### EEG Preprocessing

Although we provide the raw EEG recordings for this dataset, in order to verify the quality of the EEG signals, we followed the following preprocessing steps.

After recording, data were first downsampled to 512 Hz before running the PREP pipeline^34^ to reference the data to a robust common average. The data were then band-pass filtered between 1–30 Hz with a zero-phase Butterworth filter using *MNE*^35^.

We then extracted epochs starting one second before the beginning of each clip and lasting 5 seconds. To perform artifact correction, we used *Autoreject*^36^ on these epochs prior to fitting an independent component analysis (ICA) estimator using only the epochs marked as good by *Autoreject*. Although the duration of the epochs fed to *Autoreject* and the ICA estimator does not cover the full clip duration, this shorter time span focuses on the beginning of the clip, where one can expect eye movements and blinks to be more prominent than in later parts of the clip while transitioning from “ambient” to “focal” scene viewing^37,38^, while still providing sufficient data for threshold calculation by *Autoreject* and stable ICA decomposition. We only removed eye-related components from the continuous EEG recordings, which we identified using *ICLabel*^39^. Results from *ICLabel* were manually confirmed for a random selection of participants. All data were visually checked before and after processing.

To compute the power spectral density (PSD), we extracted epochs time-locked to the start of each clip, beginning 1 second before the clip started and ending 10 seconds after the start of the clip (so each epoch includes the neural signals corresponding to the viewing of that clip). The *Autoreject* method^36^ was used again to remove bad epochs and interpolate bad channels.

For ERP analyses, we extracted epochs beginning 200 ms before the start of the clip and comprising the first second of the clip. Again, *Autoreject* was used on these epochs.

## Data Records

The data produced during the experiment are freely accessible and can be accessed from the OpenNeuro platform via 10.18112/openneuro.ds006142.v1.0.2^32^.

Following OpenNeuro’s requirements, the dataset has been structured according to the Brain Imaging Data Structure (BIDS) standard^40,41^. The conversion from the Biosemi .bdf files in which the data were originally recorded into the BIDS standard was done in Python using the MNE-BIDS package^42^.

Within the repository, following the BIDS standard, files are organised by participant, with one folder per participant. EEG recordings have been stored following the European Data Format (.edf). Corresponding event annotations and channel information are saved in .tsv files. The event annotations include the name of the clip that was shown to participants at each point (*stim_file* column of the event .tsv files). Since we do not own the original dataset with the movie clips, we are not able to release the videos with the dataset. Instead, the *stimuli* folder contains empty text files that can be replaced with the original videos (which can be requested from the original authors^31^), although this is not required for using this dataset.

Finally, the answers from each participant to the *Qualtrics* survey are also available under the *derivatives/survey* folder. Each row represents one participant, linked to their data by the identifier in the first column of the *qualtrics_export.xlsx* file. The questions from the third section of the survey (and possible answers) are also found in this folder, in the *Questions from section 3.pdf* file.

## Technical Validation

We divide this section into two different parts: first, in the Behavioural analysis Section, we confirm that there are no significant differences in the behaviours of our two cohorts of participants. Then, in the EEG validation Section, we look at measures of the quality of the EEG signals to ensure they are viable for analyses.

### Behavioural Analysis

Given that the data were collected at two separate time points, we first compared the responses across the two cohorts to ensure that there had been no shifts in the behaviour of our participants.

**Fig. 3:**
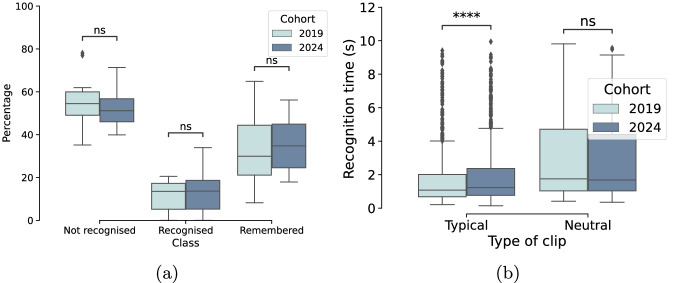
(**a**) Percentages of clips not recognised, recognised, and remembered for each cohort. (**b**) Distributions of recognition times for recognised and remembered clips, separated by cohort and type of clip. Only the first click from each participant for each video is used to calculate these distributions. Asterisks represent the statistical significance of two-sided Mann-Whitney tests after Bonferroni correction. Legend: ns: *p* > 0.05; ^****^ *p* < = 10^−4^.

We analysed differences in the percentages of clips that were remembered, recognised, and not recognised across the 2019 and 2024 cohorts as a result of the additional participant exclusion criteria and updated clip selection algorithm for the 2024 cohort. As expected, these changes resulted in a higher percentage of clips shown belonging to movies that the participants had previously watched (52% in 2019; 62% in 2024) while keeping the same experiment length (154 clips on average were shown to participants in 2019; 153 in 2024). In Fig. 3(a) we present the percentages of clips belonging to each of the three categories across both cohorts. The percentages of not recognised clips decreased slightly in 2024 (as intended by the change in clip allocation to participants), accompanied by an increase of remembered clips. However, these differences were not significant across cohorts according to two-sided Mann-Whitney tests.

We further investigated the time to the first click recorded by participants the second time they watched a recognised or remembered clip — while all times are recorded in the EEG files, only the first time for each clip is referred to as “recognition time”. In Fig. 3(b) we show the distributions of recognition times separately for each cohort and type of clip. Recognition times for “typical” clips were significantly faster for the 2019 than the 2024 cohort (two-sided Mann-Whitney *U* = 307200, *p* = 1.6 × 10^−6^), but there were no significant differences across both cohorts for “neutral” clips (*U* = 4224, *p* = 0.7). The significance was lost when we instead looked at the average participant recognition times across cohorts for each type of clip (*U*_*typical*_ = 63, *p* = 0.2; *U*_*neutral*_ = 92, *p* = 0.6). Recognition times were faster for “typical” (median = 1.15 s) than “neutral” (median = 1.70 s) clips (two-sided Mann-Whitney *U* = 112232, *p* = 2 × 10^−10^).

Finally, a Spearman test confirmed a modest, but significant correlation between clip memorability score and its recognition time (*ρ* = −0.139, *p* = 1.4 × 10^−9^), supporting the observations from the original annotators of the dataset^31^.

### EEG Validation

The quality of the EEG signals was assessed when placing the electrodes by measuring the voltage offsets from Biosemi’s ActiView programme. The voltage offsets were monitored to ensure they remained within  ±20 *μV*. Artefacts in the EEG signals were monitored during data acquisition.

#### EEG epoch rejection

**Fig. 4:**
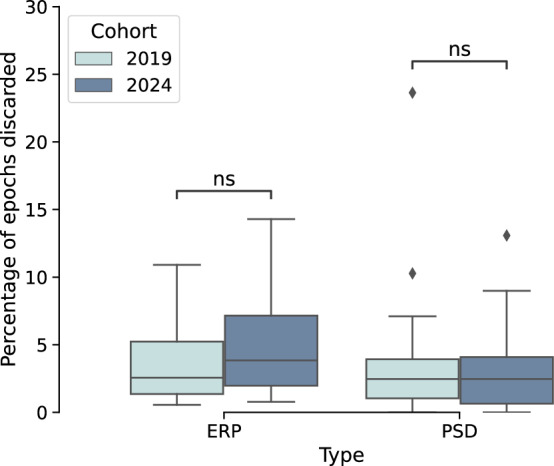
Percentages of epochs rejected from each participant for ERP and PSD analyses, separately for each cohort. Annotations represent the statistical significance of a two-sided Mann-Whitney test after Bonferroni correction. Legend: ns: *p* > 0.05.

Figure 4 compares the percentages of epochs rejected from each participant for ERP and PSD analyses, separately for each cohort. No significant differences were found in the percentages of epochs rejected across cohorts.

For frequency analyses, an average of 3.9% of epochs were rejected across participants through the pipeline described in the EEG Preprocessing Section, resulting in an average of 147.8 ± 18.5 epochs/participant remaining after rejection (range = 111–180 epochs/participant).

Numbers for epochs dropped for our ERP analyses were similar to those from the frequency analyses, with approximately 4.6% of the epochs per participant being discarded. Consequently, participants had an average of 146.7 ± 17.3 epochs left, with a range spanning from 109 to 179 epochs per participant.

#### Event-related potentials

**Fig. 5:**
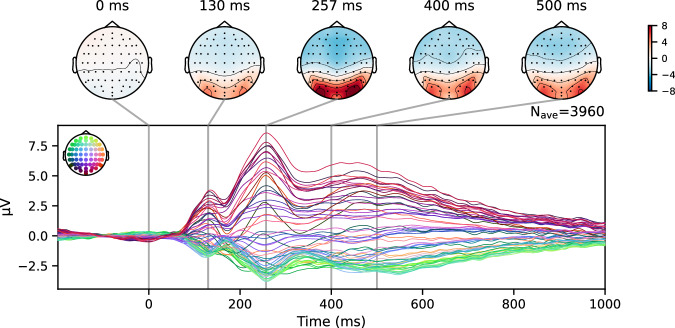
Grand averaged event-related potential time-locked to the start of a clip. *N*_*ave*_ represents the number of trials over which the channel data were averaged.

Figure 5 shows the grand averaged ERP with respect to the start of a clip presentation. Individual epochs were baselined to the average of the 200 ms-long period before the clip started playing. This ERP is consistent with those found in the literature after a cut occurs^43,44^ and confirms the correct synchronisation between the EEG triggers and the stimuli shown on the screen.

#### Power spectral density

**Fig. 6:**
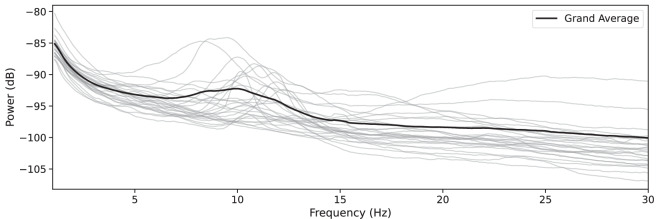
Power spectral density during clip presentation. Each line represents the average for a participant, calculated across their epochs and all channels. The black bold line represents the average across all participants.

We computed the PSD for each participant calculated over the 10-second long epochs (i.e., covering the full clip duration) and averaged across epochs and channels (thin lines) and the grand average PSD across all participants (black bold line, Fig. 6). As expected for EEG power spectra, we observed a clear peak in the alpha frequency band across participants, consistent with the characteristic spectral profile reported in the literature^45^. The variability in the individual peak alpha frequencies is reflected in the width of the peak seen in the grand average. Furthermore, there is minimal evidence of noise or artifacts (e.g., excessive power at low or high frequencies), suggesting that the recordings reliably capture underlying neural activity.

## Data Availability

The dataset is available at 10.18112/openneuro.ds006142.v1.0.2.

## Code availability

The code directory of the dataset includes an example script to load the EEG data into Python using the MNE^35^ and MNE-BIDS^42^ packages.

The custom code used to validate the dataset and generate the plots in this manuscript can be requested from the authors.
